# Single Nucleotide Polymorphisms in the G-Protein Coupled Receptor Kinase 5 (*GRK5*) Gene are associated with Plasma LDL-Cholesterol Levels in Humans

**DOI:** 10.1038/s41598-018-26055-7

**Published:** 2018-05-17

**Authors:** Stefan Z. Lutz, Mathias Falcenberg, Fausto Machicao, Andreas Peter, Martin Kächele, Elko Randrianarisoa, Angela Lehn-Stefan, Robert Wagner, Jürgen Machann, Fritz Schick, Martin Heni, Axel Ullrich, Andreas Fritsche, Norbert Stefan, Hans-Ulrich Häring, Harald Staiger, Konstantinos Kantartzis

**Affiliations:** 1 Department of Internal Medicine IV, Division of Endocrinology, Diabetology, Angiology, Nephrology and Clinical Chemistry, University of Tübingen, Tübingen, Germany; 2 Institute for Diabetes Research and Metabolic Diseases of the Helmholtz Center Munich at the Eberhard-Karls-University of Tübingen, Tübingen, Germany; 3 German Center for Diabetes Research (DZD), Tübingen, Germany; 4 Department of Molecular Biology, Max-Planck-Institute of Biochemistry, Martinsried, Germany; 5 Department of Radiology, Section on Experimental Radiology, University of Tübingen, Tübingen, Germany; 6 Institute of Pharmaceutical Sciences, Department of Pharmacy and Biochemistry, University of Tübingen, Tübingen, Germany

## Abstract

Genetically modified mice models suggest an important role for G-protein-coupled receptor kinase 5 (GRK5) in the pathophysiology of obesity and related disorders. We investigated whether single nucleotide polymorphisms (SNPs) in the gene encoding GRK5 affect cardiometabolic traits in humans. We genotyped 3 common SNPs in intron 1 (rs1980030, rs10466210, rs9325562) and one SNP in intron 3 (rs10886471) of *GRK5* in 2332 subjects at risk for type 2 diabetes. Total- and visceral fat mass were measured by magnetic resonance (MR) tomography and liver fat content by ^1^H-MR spectroscopy. Insulin secretion and sensitivity were estimated during an OGTT and measured during the euglycemic, hyperinsulinemic clamp (n = 498). Carriers of the minor allele of rs10466210 and rs1980030 had higher total- and LDL-cholesterol levels (p = 0.0018 and p = 0.0031, respectively, for rs10466210; p = 0.0035 and p = 0.0081, respectively, for rs1980030), independently of gender, age, BMI and lipid-lowering drugs. The effects of rs10466210 withstood Bonferroni correction. Similar associations were observed with apolipoprotein B levels (p = 0.0034 and p = 0.0122, respectively). Carriers of the minor allele of rs10466210 additionally displayed a trend for higher intima-media thickness of the carotid artery (p = 0.075). GRK5 may represent a novel target for strategies aiming at lowering LDL-cholesterol levels and at modifying cardiovascular risk.

## Introduction

The family of G-protein-coupled receptor kinases (GRKs) consists of seven serine/threonine kinases, which modulate several important intracellular signaling pathways. The main physiological action of GRKs is believed to be phosphorylation and thereby ‘desensitization’ (turn off) of G-protein-coupled receptors (GPCRs). GPCRs constitute the largest group of seven-transmembrane domain receptors, with more than 800 members, including the adrenergic, as well as several other hormone and cytokine receptors^1,2^. Phosphorylation of the activated GPCR leads to binding of β-arrestins, endocytosis of the receptor and ultimately to either receptor degradation or recycling and resensitization^3^. Other, kinase-independent, functions of GRKs have also been reported, including a role in inflammation (possibly by interacting with IκBα and inhibiting NFkB) and in regulating apoptosis^4–7^.

GRK5 was found to be most highly expressed in the heart and muscle as well as in the adipose tissue, but it is generally considered to be ubiquitously expressed in mammalian tissues^1^. High expression of GRK5 has been reported in several pathologies including cardiac hypertrophy and heart failure, hypertension, cancer, obesity and diabetes^1,8–13^. Additionally, based on previous *in vitro* and rodent data, GRK5 was suggested to be involved in the pathophysiology of atherosclerosis, though, both pro-atherogenic and anti-atherogenic activities were indicated^14–16^. The respective underlying mechanisms are still objective of intense research, however, the atherosclerosis-modulating effects are proposed to be mediated by modification of endothelial cell inflammation and desensitization of several cytokine and endothelin A and B receptors^3,16,17^. GRK5 may, thus, represent a possible target for the development of novel therapeutic strategies mitigating atherosclerosis.

The importance of GRK5 for metabolism was first shown in studies in *Grk5* knock-out mice (*Grk5*^*−/−*^). These mice displayed significantly less weight gain and decreased white adipose tissue (WAT) mass, decreased transcription of adipogenic genes and inhibited adipocyte differentiation during a high-fat diet^9,10^. Despite the decreased WAT mass, liver fat content was higher compared to wild type mice. *Grk5*^*−/−*^ mice also had higher glucose, insulin and triglyceride levels, and were more insulin resistant^10^. Data linking GRK5 with metabolic disorders in humans are sparse. A genome-wide association study found a significant relationship of the single nucleotide polymorphism (SNP) rs10886471 in intron 3 of *GRK5* with type 2 diabetes, but this was confined to Chinese Hans. The risk allele of rs10886471 was associated with higher *GRK5* mRNA expression and higher insulin, but not with higher glucose levels^11^. A subsequent study in another Chinese population found that the SNP rs10886471 in *GRK5* can also act as a short tandem repeat (STR) polymorphism, with the intronic (CA)_16_ allele being associated with an increased, and all other (CA)_15_ to (CA)_19_ alleles with a decreased prediabetes and type 2 diabetes risk^12^.

Nevertheless, despite the diversity of metabolic disorders that occur in *Grk5*^*−/−*^ mice, it was not investigated whether the SNP rs10886471 (or any other SNP in *GRK5*) has effects on metabolism other than those on glucose and insulin levels in any of the aforementioned studies^11,12^. In the present study we, therefore, explored possible associations of selected SNPs in *GRK5* gene with body fat mass and distribution, as well as with other relevant traits of glucose and lipid metabolism, in a large cohort of phenotypically well-characterized Caucasians who were at risk for type 2 diabetes. Three tagging SNPs covering the first 4 kb of intron 1, a region highly enriched for gene-regulatory elements, and the SNP rs10886471, which was reported to be related with type 2 diabetes in Chinese Hans^11^ in intron 3 of *GRK5* were genotyped and included in the analyses.

## Research Design and Methods

### Subjects

We analyzed data of 2332 unrelated Caucasians, 1469 women and 863 men, from the southern part of Germany, who participated in an ongoing study on the pathophysiology and prevention of type 2 diabetes^18–21^. Inclusion criteria were as follows: a family history of type 2 diabetes, a body mass index (BMI) ≥ 27 kg/m^2^, previous diagnosis of impaired glucose tolerance or of gestational diabetes. All subjects were considered healthy according to a physical examination and routine laboratory tests. As assessed by means of a standard questionnaire, they had no history of liver disease and did not consume more than 2 alcoholic drinks per day. Serum aminotransferase levels were lower than 3 times the upper limit of normal.

From the subjects who met the aforementioned requirements, mostly due to technical reasons, a subgroup of 339 (210 women and 129 men) had measurements of body fat distribution and 455 (292 women and 163 men) measurements of liver fat content using magnetic resonance techniques. All subjects underwent an oral glucose tolerance test and a subgroup (n = 498, 263 women and 235 men) additionally a euglycemic, hyperinsulinemic clamp. Informed written consent was obtained from all participants and the Ethics Committee of the University of Tübingen had approved the protocol according to the Helsinki Declaration.

### Body fat mass and body fat distribution

The BMI was calculated as weight divided by the square of height (kg/m^2^). Waist circumference was measured at the midpoint between the lateral iliac crest and the lowest rib. Total adipose tissue (TAT) and visceral adipose tissue (VAT) mass were measured by magnetic resonance (MR) tomography, with an axial T1-weighed fast spin echo technique with a 1.5 T whole-body imager (Magnetom Sonata, Siemens Healthcare) as previously described^22^.

### Liver fat content

Liver fat content was measured by localized ^1^H-MR spectroscopy, as previously described^23^.

### Oral glucose tolerance test (OGTT)

All individuals underwent a 2-hour 75-g OGTT. Venous plasma samples were obtained at 0, 30, 60, 90 and 120 minutes for determination of plasma glucose and insulin levels. Blood glucose was determined using a bedside glucose analyzer (glucose-oxidase method; YSI, Yellow Springs Instruments, Yellow Springs, OH, USA). For all other measurements, blood was placed on ice immediately after drawing and transferred to the lab for subsequent centrifugation and analysis. Plasma insulin and C-peptide concentrations were determined by a chemiluminescent immunoassay (ADVIA Centaur XP, Siemens Healthcare Diagnostics, Eschborn, Germany).

Insulin sensitivity was estimated from the OGTT as proposed by Matsuda and DeFronzo [10000/√(Ins_mean_∙Glc_mean_∙Ins_0’_∙Glc_0’_), with Ins = insulin and Glc = glucose]^24^. Furthermore, the homeostasis model insulin resistance index (HOMA-IR) = Ins_0’_ [in μU/ml]∙Glc_0’_ [in mmol/l]/22.5 was calculated^25^.

OGTT-derived insulin secretion was calculated as the insulinogenic index [IGI, (Ins_30’_-Ins_0’_)/(Glc_30’_-Glc_0’_)] and area under the curve (AUC) C-Pep_0’−120’_/AUC Glc_0’−120’_, with C-Pep = C-peptide. The latter was calculated according to the trapezoid method as ½[½(C-Pep_0’_) + C-Pep_30’_ + C-Pep_60’_ + C-Pep_90’_ + ½(C-Pep_120_)]/½[½(Glc_0’_) + Glc_30’_ + Glc_60’_ + Glc_90’_ + ½(Glc_120’_)]. Both indices were previously shown to be well-suited to detect genetically determined impaired β-cell function^26^.

### Euglycemic, hyperinsulinemic clamp

A subgroup of subjects (n = 498) additionally underwent a euglycemic, hyperinsulinemic clamp. Insulin sensitivity was determined with a primed insulin infusion at a rate of 40 mU∙m^−2^∙min^−1^ for 2 hours as previously described^27^. Insulin sensitivity index from the clamp (ISI_clamp_) was calculated as glucose infusion rate necessary to maintain euglycemia during the last 20 min (steady state) of the clamp (in μmol/kg/min) divided by the steady-state insulin concentration (in pmol/l).

### Carotid intima-media thickness

Intima-media thickness of the carotid artery was measured by high resolution ultrasound. (AU5 idea, Esaote Biomedica, Munich, Germany) with an integrated electrocardiography (ECG) package as previously described^28^.

### Laboratory measurements

Glycated haemoglobin (HbA1c) measurements were performed in the central laboratory of the University Hospital of Tübingen using the Tosoh A1c analyzer HLC-723G8 (Tosoh Bioscience GmbH, Griesheim, Germany). Plasma free fatty acid (FFA) concentrations (enzymatic method, WAKO Chemicals, Neuss, Germany) as well as clinical chemical measurements (total-, HDL- and LDL-cholesterol, triglycerides) were measured on the ADVIA XPT Clinical Chemistry System (Siemens Healthcare Diagnostics, Eschborn, Germany). Serum concentrations of apolipoproteins were determined on the BN ProSpecnephelometer (Siemens Healthcare Diagnostics, Eschborn, Germany).

### Selection of tagging SNPs and genotyping

We focused on the first 4 kb of intron 1 of the human *GRK5* gene on chromosome 10, due to the high density of gene-regulatory elements (promoter and enhancer elements) in this gene region (according to data of the 1000 Genome Project http://www.internationalgenome.org/1000-genomes-browsers). There are no other promoter regions in the gene, neither upstream nor downstream of the promoter. There are several putative promoter flanking sequences downstream of the GRK5 gene promoter, which have probably no significant relation to the core promoter (Supplemental Figs 1 and 2).

**Figure 1:**
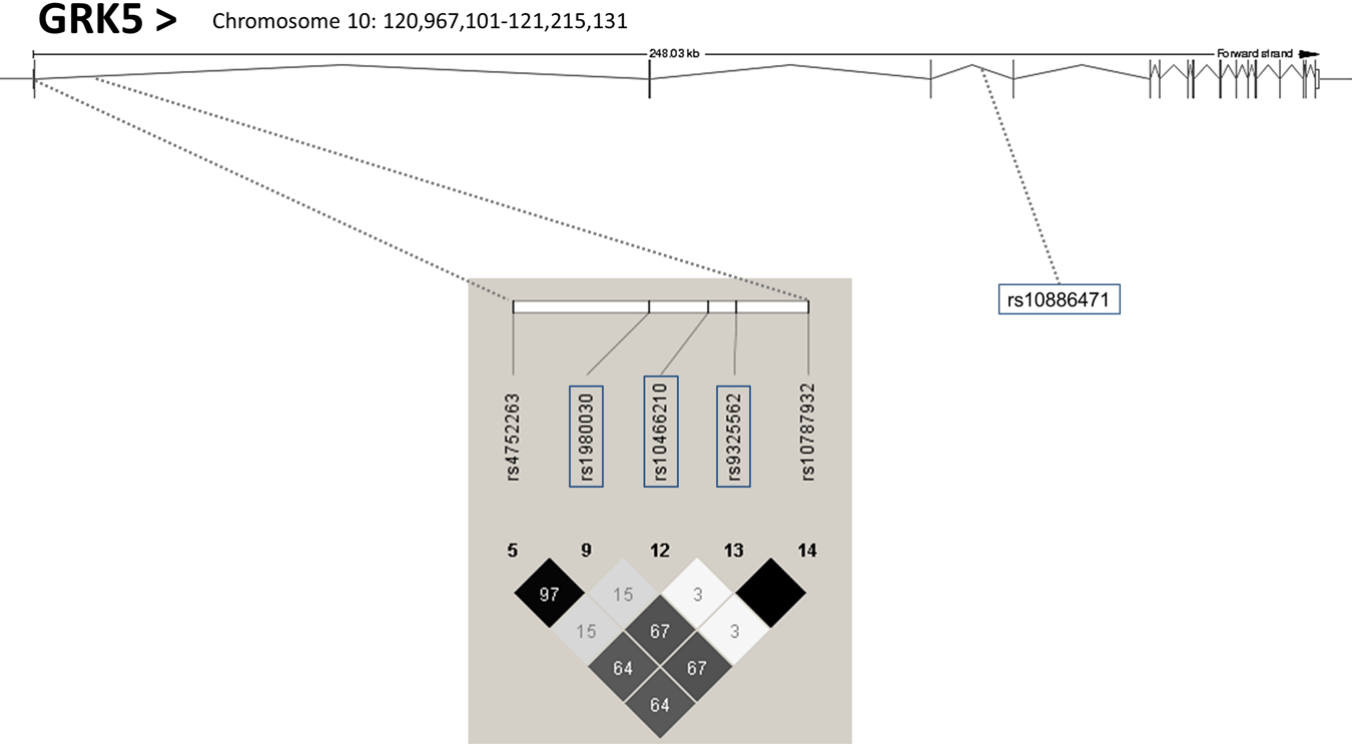
Genomic region of human chromosome 10 harboring the *GRK5* gene. The HapMap linkage disequilibrium (r^2^) data of the 5 common (MAF ≥ 0.05) SNPs in intron 1 are shown. Since rs4752263 and rs190030, as well as rs10787932 and rs9325562, were in almost complete linkage disequilibrium, only the second member of the each pair was genotyped (framed). In addition the position of another common SNP (rs10886471) in intron 3 of the gene is depicted.

Based on publicly available phase II data of the International HapMap Project derived from Utah residents with Central European ancestry (release #24 November 2008, http://hapmap.ncbi.nlm.nih.gov/index.html.en), we identified 5 common SNPs (minor allele frequencies ≥0.05) in this intronic gene region, i.e., rs4752263, rs1980030, rs10466210, rs9325562, and rs10787932. Since rs4752263 and rs1980030, and also rs9325562 and rs10787932 were in linkage disequilibrium (r^2^ = 0.97 and r^2^ = 1, respectively) according to HapMap data, only the SNPs rs1980030, rs10466210 and rs9325562 were genotyped and further analysed. The HapMap linkage disequilibrium (r^2^) data of these five common SNPs are schematically shown in Fig. 1. Additionally, we genotyped rs10886471 in intron 3 of the *GRK5* gene, because it was found in a genome-wide association study to be significantly associated with type 2 diabetes in Chinese Hans^11^. We could not find any data regarding LD among rs10886471 and the other SNPs in Europeans.

For genotyping, DNA was isolated from whole blood using a commercial DNA isolation kit (NucleoSpin, Macherey & Nagel, Düren, Germany). The 4 SNPs were genotyped using the Sequenom mass ARRAY system with iPLEX software (Sequenom, Hamburg, Germany). The genotyping success rates were 99.7%. The Sequenom results were validated by bidirectional sequencing in 50 randomly selected subjects, and both methods gave 100% identical results.

### Statistical analyses

Since most of the variables were non-normally distributed, data are given as counts or medians [interquartile range]. For statistical analysis, data that were not normally distributed (Shapiro-Wilk *W* test) were logarithmically transformed. The univariate interrelationships (Pearson correlations) between the phenotypes tested are given in supplemental Table 1. To identify independent associations of the SNPs, we performed multiple linear regression analyses with the relevant metabolic traits set as dependent variables. BMI, waist circumference, TAT mass, VAT mass and liver fat content were adjusted for age and gender. The other traits were additionally adjusted for BMI. Lipid measurements (FFA, triglycerides, total-, HDL- and LDL-cholesterol levels, and apolipoproteins) were also adjusted for treatment with lipid-lowering medication and insulin secretion measurements also for insulin sensitivity (as estimated from the OGTT). The genotype was included in the analyses as an independent variable. For each dependent variable two models were applied. In the additive inheritance model, the effects of all genotypes on the dependent variable were compared and allele-dose effects were tested; in the dominant model, homozygotes for the major allele were compared to heterozygotes and homozygotes for the minor allele combined. The Hardy-Weinberg equilibrium was tested using a χ^2^-test (1 degree of freedom). The statistical software package JMP 11.0 (SAS Institute Inc, Cary, NC, USA) was used. We performed Bonferroni correction for the 4 SNPs tested and the following 4 physiologically unrelated traits: BMI (as being closely associated with body weight, waist circumference, total body, visceral and liver fat), insulin sensitivity and insulin secretion (as being strongly related to glucose, C-peptide and insulin levels, as well as HDL-cholesterol and triglyceride levels), and LDL-cholesterol, for the additional reason of being causally related to intima-media thickness, while it is physiologically not directly linked to insulin sensitivity. Accordingly, a p-value ≤ 0.0032 (Bonferroni corrected for 4 × 4 = 16 null hypotheses) was considered statistically significant. Associations with a p-value between 0.0032 and 0.05 were considered nominal.

### Availability of materials and data

The datasets used and/or analysed during the current study are available from the corresponding author on reasonable request.

## Results

### Demographic, anthropometric and metabolic characteristics of the subjects

The characteristics of the whole population, i.e. the 2332 subjects, are shown in the Table 1. All measurements covered a wide range, e.g. age 18–82 years, body weight 42–244 kg, BMI 16.30–86.45 kg/m^2^, waist circumference 38–185 cm, TAT mass 9.98–56.32 kg, VAT mass 0.36–8.90 kg, liver fat content 0.16–30.11%, fasting FFA 80–3756 μM, 2-hour FFA 10–1497 μM, total cholesterol 63–468 mg/dl, LDL-cholesterol 15–295 mg/dl, HDL-cholesterol 24–138 mg/dl, triglycerides 15–3202 mg/dl, apolipoprotein AI 54–316 mg/dl, apolipoprotein B 37–187 mg/dl, insulin sensitivity 2.19–85.37 10^19^ l^2^∙mol^−2^ as assessed by the OGTT, 0.008–0.472 10^6^ l∙kg^−1^∙min^−1^ as measured by the clamp.

**Table 1:** Characteristics of the whole study population (n = 2332).

n (females/males)	1469/863
Age (years)	40.0 [29.0–51.0]
BMI (kg∙m^−2^)	28.64 [24.24–35.85]
Waist circumference (cm)	96.0 [84.0–110.0]
Total body fat mass _MRT_ (% BW)^*^	30.23 [23.52–37.82]
Visceral fat mass _MRT_ (% BW)^*^	3.14 [1.89–4.30]
Liver fat _MRS_ (%)**	3.37 [1.56–8.09]
Fasting glucose (mM)	5.11 [4.83–5.50]
2 h glucose _OGTT_	6.22 [5.22–7.28]
Fasting Insulin (pM)	60 [39–102]
2 h Insulin (pM)	360 [202–668]
Fasting free fatty acids (μM)	570 [429–724]
2 h free fatty acids (μM)	73 [47–113]
Fasting triglycerides (mg/dl)	102.0 [72.0–147.0]
Total cholesterol (mg/dl)	190.0 [166.0–216.0]
HDL-cholesterol (mg/dl)	51.0 [43.0–61.0]
LDL-cholesterol (mg/dl)	116.0 [96.0–139.0]
Apolipoprotein AI (mg/dl)	152.0 [134.0–171.0]
Apolipoprotein B (mg/dl)	90.0 [75.0–107.0]
Glycated hemoglobin - HbA1c (%)	5.40 [5.10–5.70]
HOMA-IR index (10^–6^ mol∙U∙l^−2^)	2.31 [1.42–4.03]
Insulin sensitivity _OGTT_ (10^19^ l^2^∙mol^−2^)	10.65 [6.33–17.38]
ISI_clamp_ (10^6^ l∙kg^−1^∙min^−1^)^***^	0.074 [0.051–0.111]
C-peptide 30’(pmol/l)	1889 [1434–2489]
IGI [(Ins_30′_-Ins_0′_)/(Glc_30′_-Glc_0′_)]	140.7 [86.3–226.6]
AUC C-Pep _′0−30′_/AUC Glc_0′−30′_	184.6 [143.6–239.1]
AUC C-Pep _0′−120′_/ AUC Glc_0′−120′_	298.8 [242.9–364.7]

^*^Available in 339 subjects; ^**^available in 455 subjects; ^***^available in 498 subjects.

Data are given as counts or medians [interquartile range]. BMI, body mass index; BW, body weight; OGTT, oral glucose tolerance test; HOMA-IR, homeostasis model assessment of insulin resistance; ISI, insulin sensitivity index; IGI, insulinogenic index; Ins, insulin; C-Pep, C-peptide; Glc, glucose; AUC, Area Under the Curve.

### Genotyping of *GRK5* tagging SNPs

The 2332 study participants were genotyped for the three SNPs rs1980030, rs10466210, rs9325562 in intron 1, and the SNP rs10886471 in intron 3 of *GRK5*. All SNPs were in Hardy-Weinberg equilibrium (all p > 0.05). The observed MAFs were 0.440 (rs1980030), 0.072 (rs10466210), 0.356 (rs9325562), 0.467 (rs10886471), and were similar to those provided by HapMap for the Central European population (0.369, 0.081, 0.283, and 0.455, respectively). According to the HapMap data (Fig. 1), the genetic linkage between the three SNPs in intron 1 was low or moderate with minimal r^2^ = 0.03 and maximal r^2^ = 0.67.

### Associations of *GRK5* SNPs with body fat compartments and liver fat content

After adjustment for age and gender, none of the four SNPs showed significant associations with BMI, waist circumference, TAT mass or VAT mass (Table 2 and Supplemental Table 2). A nominal association of the SNP rs10466210 with VAT mass (p = 0.022 in the additive, p = 0.018 in the dominant model, Table 2) was observed. Similarly, we found no significant association of any of the SNPs with liver fat content, as measured by ^1^H-MR spectroscopy (Table 2 and Supplemental Table 2).

**Table 2:** Associations of the SNPs rs10466210 and rs1980030 in *GRK5* gene with demographic and metabolic characteristics.

Variable	rs10466210					rs1980030				
	GG	GA	AA	p add	p dom	AA	AG	GG	p add	p dom
n (females/males)	1257/753	201/107	11/3	0.19^#^	0.25^#^	452/282	728/417	289/164	0.39	0.34
Age (years)	40.0 [29.0–51.0]	40.0 [30.0–51.8]	40.0 [26.0–44.5]	0.88	0.99	40.0 [30.0–50.0]	39.0 [29.0–50.0]	40.0 [30.0–52.0]	0.61	0.61
BMI (kg•m^−2^)	28.63 [24.28–35.86]	28.40 [24.06–35.14]	35.57 [24.99–42.98]	0.81	0.92	28.37 [24.42–35.96]	28.65 [24.05–35.83]	28.90 [24.31–35.66]	0.96	0.80
Waist circumference (cm)	96.0 [84.0–110.0]	95.0 [84.0–109.0]	103.0 [85.8–119.3]	0.79	0.92	96.0 [84.0–109.0]	95.0 [83.0–109.5]	96.0 [85.0–110.1]	0.68	0.94
Total body fat mass _MRT_ (% BW)^*^	30.54 [23.53–37.92]	28.03 [23.03–37.38]	30.17	0.95	0.93	30.74 [24.22–38.02]	30.66 [22.91–38.29]	29.82 [23.73–35.76]	0.49	0.88
Visceral fat mass _MRT_ (% BW)^*^	3.17 [1.90–4.30]	2.49 [1.71–4.32]	3.84	0.022	0.018	3.05 [1.88–4.19]	3.17 [1.91–4.30]	3.24 [1.88–4.60]	1.00	0.89
Liver fat _MRS_ (%)^**^	3.43 [1.61–8.16]	3.27 [1.26–6.39]	1.89 [0.68–3.10]	0.14	0.19	3.50 [1.49–8.15]	3.26 [1.57–8.07]	3.60 [1.64–8.06]	0.69	0.79
Fasting glucose (mM)	5.11 [4.83–5.53]	5.11 [4.83–5.50]	5.20 [4.89–5.51]	0.91	0.78	5.11 [4.83–5.51]	5.11 [4.80–5.54]	5.11 [4.83–5.50]	0.96	0.93
2 h glucose _OGTT_ (mM)	6.22 [5.22–7.28]	6.22 [5.17–7.28]	6.17 [5.56–7.56]	0.63	0.69	6.22 [5.28–7.29]	6.17 [5.17–7.22]	6.33 [5.28–7.39]	0.91	0.48
Fasting Insulin (pM)	60 [38–101]	61 [40–108]	76 [37–181]	0.30	0.32	59 [38–100]	60 [38–101]	63 [40–108]	0.21	0.43
2 h Insulin (pM)	358 [201–663]	378 [211–687]	590 [296–1021]	0.18	0.21	359 [199–673]	353 [198–647]	377 [218–690]	0.22	0.60
Fasting free fatty acids (μM)	566 [429–721]	605 [428–748]	603 [446–872]	0.11	0.11	559 [417–722]	570 [430–722]	585 [445–745]	0.13	0.22
2 h free fatty acids (μM)	73 [46–112]	75 [48–124]	77 [54–125]	0.029	0.026	74 [45–113]	72 [47–115]	74 [48–112]	0.32	0.34
Fasting triglycerides (mg/dl)	101.0 [72.0–148.0]	107.5 [70.3–146.0]	118.0 [82.5–150.5]	0.39	0.39	98.0 [71.0–146.3]	104.0 [72.0–150.0]	103.0 [75.0–146.0]	0.057	0.041
Total cholesterol (mg/dl)	188.0 [166.0–216.0]	195.5 [174.3–221.0]	201.0 [178.5–219.5]	**0.0018**	**0.0019**	186.0 [164.5–214.0]	190.0 [167.0–217.0]	194.0 [168.0–222.0]	0.0035	0.0037
HDL-cholesterol (mg/dl)	51.0 [43.0–61.0]	52.0 [43.0–63.0]	49.0 [41.5–54.5]	0.18	0.13	51.0 [43.0–61.0]	52.0 [43.0–62.0]	51.0 [43.0–62.0]	0.77	0.99
LDL-cholesterol (mg/dl)	115.0 [95.0–138.0]	122.0 [101.0–142.0]	132.0 [116.0–160.5]	**0.0031**	0.0058	113.0 [94.0–134.0]	116.5 [96.0–140.0]	121.5 [100.0–140.0]	0.0081	0.036
Apolipoprotein AI (mg/dl)	152.0 [134.0–170.0]	154.0 [135.3–175.8]	142.0 [133.0–147.0]	0.22	0.12	152.0 [135.0–170.0]	152.0 [135.0–173.0]	150.5 [133.0–168.3]	0.40	0.77
Apolipoprotein B (mg/dl)	89.0 [75.0–107.0]	93.0 [81.0–111.0]	102.0 [83.0–113.0]	0.0034	**0.0029**	88.0 [73.0–105.0]	90.0 [76.0–107.0]	93.0 [77.0–110.0]	0.0122	0.032
Glycated hemoglobin - HbA1c (%)	5.40 [5.10–5.70]	5.50 [5.20–5.80]	5.50 [5.05–5.85]	0.0088	0.0057	5.40 [5.10–5.70]	5.40 [5.10–5.70]	5.40 [5.10–5.80]	0.28	0.18
HOMA-IR index (10^−6^ mol•U•l^−2^)	2.31 [1.41–4.00]	2.34 [1.44–4.22]	2.66 [1.40–7.74]	0.33	0.33	2.31 [1.40–3.91]	2.29 [1.41–4.04]	2.41 [1.45–4.17]	0.24	0.49
Insulin sensitivity _OGTT_ (10^19^ l^2^•mol^−2^)	10.70 [6.37–17.49]	10.60 [6.27–17.19]	10.62 [3.00–13.99]	0.41	0.45	10.84 [6.34–17.58]	10.78 [6.42–17.91]	10.09 [6.17–16.87]	0.36	0.65
ISI_clamp_ (10^6^ l•kg^−1^•min^−1^)^***^	0.074 [0.048–0.112]	0.073 [0.053–0.107]	0.058	0.66	0.71	0.076 [0.051–0.113]	0.073 [0.049–0.110]	0.073 [0.051–0.111]	0.67	0.18
C-peptide 30’(pmol/l)	1878 [1428–2483]	1951 [1456–2565]	2073 [1560–2673]	1.00	0.86	1893 [1436–2483]	1875 [1407–2524]	1938 [1488–2465]	0.45	0.46
IGI [(Ins_30‘_-Ins_0’_)/(Glc_30‘_-Glc_0’_)]	139.7 [85.5–226.6]	148.4 [90.1–230.5]	162.2 [118.0–209.9]	0.32	0.19	140.7 [86.6–226.2]	142.7 [86.3–232.7]	132.9 [84.1–209.5]	0.17	0.88
AUC C-Pep_0‘−30’_/AUC Glc_0‘−30’_	183.5 [143.4–239.0]	191.8 [144.4–239.5]	192.6 [154.3–249.8]	0.94	0.78	183.1 [144.4–242.1]	185.3 [142.5–239.9]	184.8 [147.6–235.1]	0.28	0.41
AUC C-Pep_0‘−120’_/AUC Glc_0‘−120’_	298.6 [241.9–364.6]	301.4 [245.0–366.8]	305.3 [248.9–376.4]	0.82	0.88	293.2 [245.1–367.3]	300.2 [240.0–367.0]	302.2 [245.6–361.7]	0.60	0.81

^*^Available in 339 subjects; ^**^available in 455 subjects, ^***^available in 498 subjects.

Values represent means ± SE (standard error). For statistical analyses, non-normally distributed variables were log transformed. The genotype effect was tested using additive and dominant inheritance models. BMI, waist circumference, total body fat mass, visceral fat mass and liver fat were adjusted for age and gender. Glucose and insulin levels, glycated hemoglobin, and insulin sensitivity measures (HOMA-IR, insulin sensitivity from the OGTT and ISI_clamp_) were additionally adjusted for BMI. Lipid measurements (free fatty acids, total-, HDL- and LDL-Cholesterol, and triglycerides) were further adjusted for lipid-lowering medication. Insulin secretion measures (C-peptide 30’, IGI, AUC C-peptide 0-30 / AUC Glc 0-30, AUC C-peptide 0-120/ AUC Glc 0-120) were adjusted for gender, age, BMI and insulin sensitivity from the OGTT. #χ^2^-test. BW, body weight; ISI, insulin sensitivity index; IGI, insulinogenic index; Ins, insulin; C-Pep, C-peptide; Glc, glucose; AUC, Area Under the Curve.

### Associations of *GRK5* SNPs with glycemia, insulin sensitivity and insulin secretion

Neither fasting nor 2-hour post OGTT glycemia and insulinemia, or insulin sensitivity assessed by the HOMA-IR or during the OGTT, or measured by the clamp (adjusted for gender, age and BMI) were associated with any of the four SNPs. We observed a nominally significant association of the SNP rs10466210 with glycated hemoglobin: carriers of the minor A-allele had higher glycated hemoglobin levels (p = 0.0088 in the additive, p = 0.0057 in the dominant model, Table 2). However, no other SNP was significantly related to glycated hemoglobin levels. Likewise, the four SNPs were not associated with adjusted insulin secretion (Table 2 and Supplemental Table 2).

### Associations of *GRK5* SNPs with plasma lipids

**Figure 2:**
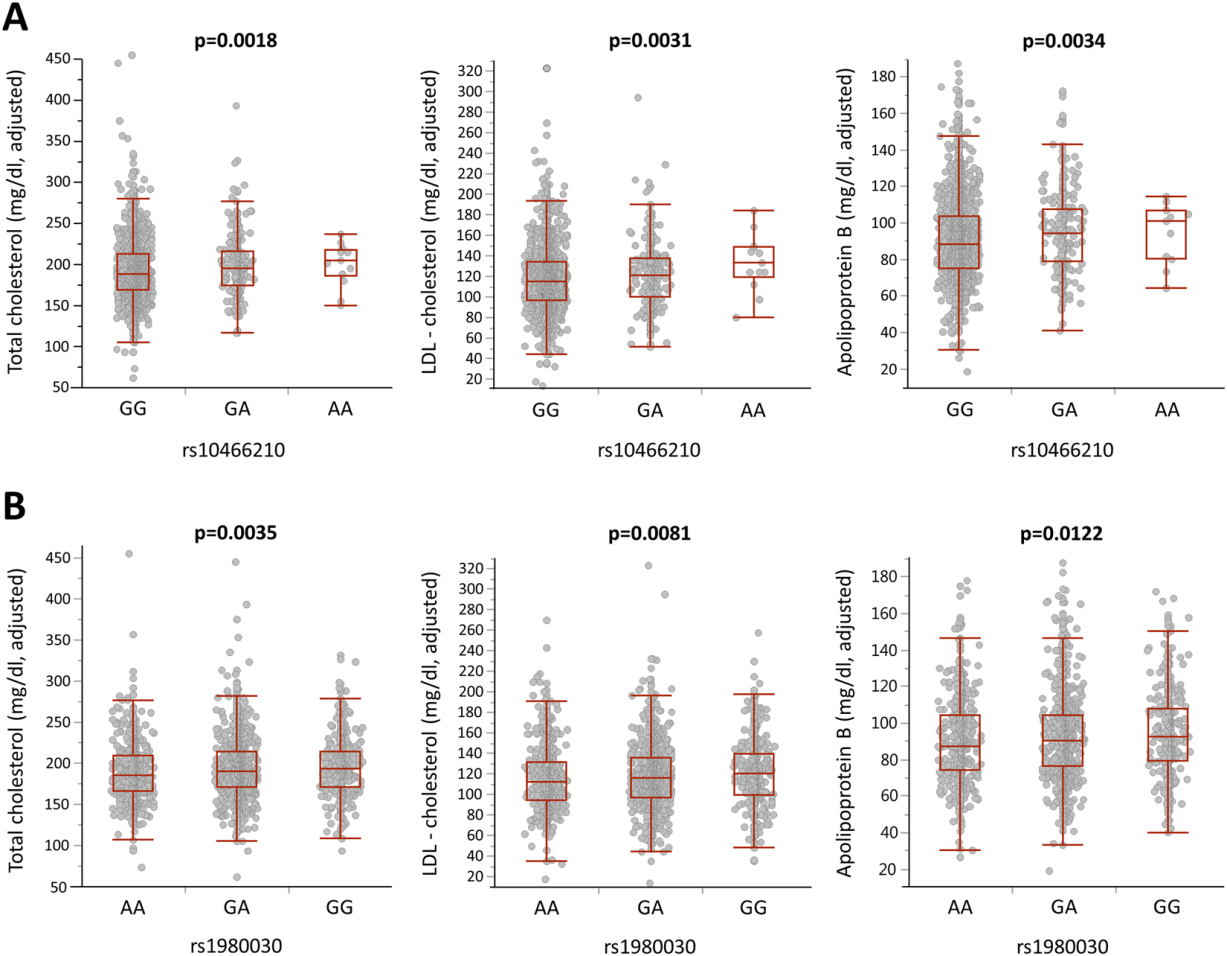
Associations of total- and LDL-cholesterol, as well as apolipoprotein B levels with rs10466210 (**A**) and rs1980030 (**B**) SNPs in *GRK5* gene in 2332 subjects. For statistical analyses, all lipid measurements were log-transformed and adjusted for gender, age, BMI and lipid-lowering medication. Adjusted back-transformed values and box blots (showing median sample values and the 75^th^ and 25^th^ quantiles) are depicted.

**Figure 3:**
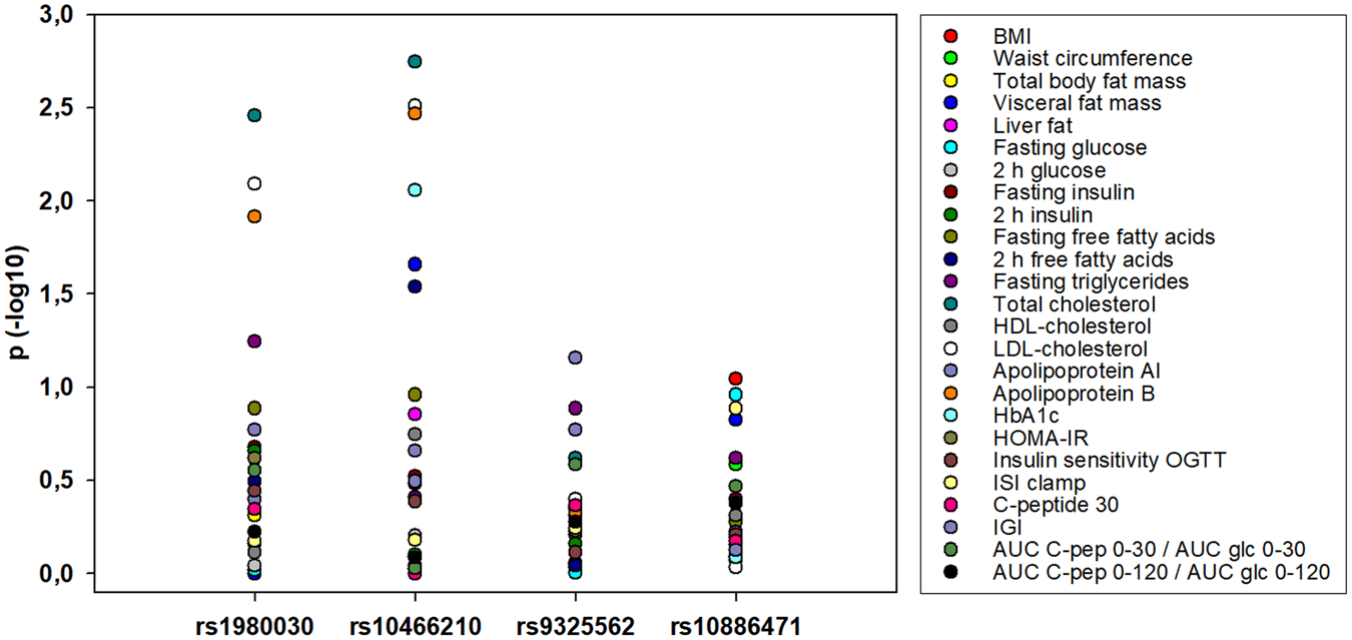
Manhattan plot of the phenome-wide association results. ISI, insulin sensitivity index; IGI, insulinogenic index; C-pep, C-peptide; glc, glucose; AUC, Area Under the Curve.

In contrast to the absence of any association or only rather sporadic associations of the *GRK5* SNPs with body fat mass and body fat distribution, as well as glucose homeostasis, we found robust associations of two SNPs, rs10466210 and rs1980030, with total- and LDL-cholesterol, and apolipoprotein B levels, which is known to be the main protein of LDL-particles, but not with HDL-cholesterol or triglyceride levels (Table 2 and Fig. 2). In particular, the minor A-allele of rs10466210 was significantly associated with higher total cholesterol (p = 0.0018 in the additive and p = 0.0019 in the dominant model), LDL-cholesterol (p = 0.0031 in the additive model) and apolipoprotein B (p = 0.0034 in the additive and p = 0.0029 in the dominant model) levels, withstanding Bonferroni correction in all instances. The minor G-allele of rs1980030 was also related to higher total cholesterol, LDL-cholesterol and apolipoprotein B concentrations, but the associations fell very short from being significant after Bonferroni correction for total cholesterol (p = 0.0035 in the additive model and p = 0.0037 in the dominant model) and were only nominally significant for LDL-cholesterol and apolipoprotein B (p = 0.0081 and p = 0.0122, respectively in the additive model, and p = 0.036 and p = 0.032, respectively in the dominant model), presumably due to the lower effect size of the SNP (Table 2). We performed *post hoc* power analyses of the associations of the SNP with the lowest minor allele frequency, rs10466210, in the additive model with total cholesterol, LDL-cholesterol and apolipoprotein B, all adjusted for gender, age, BMI and lipid-lowering medication. Based on the Bonferroni-corrected α-level of 0.0032, our study was sufficiently powered (1-β > 0.8) to detect effect sizes as small as 3 mg/dl for all three traits (1-β = 0.83 for total cholesterol, 0.93 for LDL-cholesterol and 0.99 for apolipoprotein B). The other 2 SNPs, rs9325562 and rs10886471, were not related to any of the plasma lipids or lipoproteins (Supplemental Table 2). The phenome-wide association results for all 4 SNPs are also shown as Manhattan plots (Fig. 3). Although there was no association of the SNPs with weight or BMI, and in order to exclude the possibility that the significant associations were driven by extremely obese subjects, we repeated these analyses after excluding subjects with BMI ≥ 65 kg/m^2^ (n = 14). The results (p-values) were virtually the same (Supplemental Table 3).

### Associations of *GRK5* SNPs with carotid intima-media thickness (IMT)

Having found significant associations of two SNPs with LDL-cholesterol and apolipoprotein B levels, we set out to explore whether these SNPs were associated with IMT of the common carotid artery, a known early marker of atherosclerosis. IMT measurements were available in a subgroup of 590 subjects. We observed a trend for an association of the SNP rs10466210 [GG: n = 514, IMT 0.535 (0.465–0.639) mm, median (interquartile range); GA: n = 75, IMT 0.575 (0.494–0.670) mm; AA: n = 1, IMT 0.750 mm] with IMT (p = 0.075 in the additive model and p = 0.10 in the dominant model). The minor allele A, which was significantly associated with higher total- and LDL-cholesterol, and apolipoprotein B levels in the whole study population, showed a trend for an association with higher carotid IMT in this smaller group. The SNP rs1980030 was not associated with IMT (p > 0.57 in both models), presumably due to its lower effect size.

## Discussion

In this study, we investigated possible associations of selected common SNPs in *GRK5* with body fat mass and distribution, and measurements of glucose and lipid metabolism. We found robust associations of the minor alleles of two SNPs, rs10466210 and rs1980030, with higher total- and LDL-cholesterol, as well as with higher apolipoprotein B (apoB) levels. We detected no association of either of the SNPs with HDL-cholesterol or triglyceride levels. Of particular note, the two aforementioned SNPs were not linked to each other (r^2^ = 0.15, Fig. 1).

A possible impact of GRK5 on atherosclerosis was suggested in *in vitro* and *in vivo* studies^14–16^. Our findings were somehow unexpected, because they are not completely in agreement with the results of studies in *Grk5* knock-out mice^9,10^. With regard to plasma lipids, these animals had, under a high-fat diet, higher plasma triglyceride levels, but not significantly elevated total-, HDL- or LDL-cholesterol levels. However, these animals also displayed a higher insulin resistance and a higher liver fat content^10^, which may account for the higher plasma triglyceride levels (although, one would also expect them to have lower HDL-cholesterol levels^29^). Besides, under a standard diet there was no difference in plasma triglyceride levels between knock-out and wild-type mice. In our study none of the SNPs was related with insulin resistance. Thus, it is not surprising that the SNPs were not associated with triglycerides, either. Of note, LDL-cholesterol levels were higher in *Grk5*^*−/−*^ mice, although this relationship was not statistically significant^10^. In any case, we do not believe that the data from knock-out mice can raise doubts about the robustness of our findings. In addition to the more or less expected differences between rodents and humans, the association of the two SNPs with LDL-cholesterol levels is, according to our analyses, strong, withstands correction for multiple testing (at least for rs10466210) and is virtually ‘replicated’ in the association of the SNPs with a separate measurement, apoB, which represents the main apoprotein of LDL-particles. In addition, the associations were significant independently of gender, BMI and lipid-lowering medication. In the Global Lipids Genetics Consortium the associations of the same two SNPs with total- and LDL-cholesterol levels were not significant, but adjustment was done only for age and sex, and not for BMI or medication (http://csg.sph.umich.edu/abecasis/public/lipids2013/)^30^.

It is very difficult to assume a mechanism by which GRK5 may affect LDL-cholesterol metabolism. This is of course due to the large number of GPCRs and the large diversity of their possible ligands. Furthermore, it is not known whether the two SNPs found to be associated with LDL-cholesterol and apoB levels are functional, or whether they may simply be linked to other functional SNPs. The risk alleles of the SNPs rs4752300 and rs10886471 were found to enhance *GRK5* mRNA expression in peripheral blood cells and in adipose tissue, respectively^11,31^. According to HapMap, the SNP rs1980030 is in linkage disequilibrium with the SNP rs4752300 (r^2^ = 0.97, Fig. 1).

Following our main finding of associations of the two SNPs with LDL-cholesterol and apoB levels, we further tested whether there may be significant associations of the *GRK5* SNPs with IMT, an early marker of atherosclerosis. We detected a trend for an association of the SNP rs10466210 with IMT. The reason why the association did not reach statistical significance may be the fact that measurements of IMT were only available in about a quarter of the participants. No significant association of SNP rs1980030 with IMT was found. This may be explained, in addition to the limited number of available IMT measurements, by the clearly smaller effect size of the association of this SNP with LDL-cholesterol and apoB concentrations (Table 2). Nevertheless, our findings regarding IMT point to an involvement of GRK5 in cholesterol metabolism with potential clinical relevance.

With the only exception of isolated nominal associations of the SNP rs10466210 with VAT mass and glycated hemoglobin, which probably represent chance findings, we found no significant associations of the four SNPs in *GRK5* with body fat mass and distribution and measurements of glucose metabolism. In contrast, *Grk5*^*−/−*^ mice were resistant to adipogenesis and adipocyte differentiation of WAT under a high fat diet, while they concomitantly developed fatty liver. They also had higher glucose and insulin levels, and were more insulin resistant^9,10^. Differences between the species constitute an obvious explanation for the apparent conflicting findings in knock-out mice and humans. Furthermore, since all SNPs we studied in humans were intronic, they may only slightly modulate gene expression, thereby, exerting a rather small effect on metabolism, while knocking-out of the whole gene in rodents may result in more potent and more complex effects. For instance, while insulin resistance may account for the higher insulin levels, and the higher insulin and glucose levels may account for liver fat accumulation, the reasons for insulin resistance and higher glucose levels in these animals are unknown.

To our knowledge, there are principally two studies in the literature linking GRKs with glucose metabolism in humans. The first was a genome-wide association study, which found a significant relationship of the SNP rs10886471 in *GRK5* with type 2 diabetes. The risk allele was associated with higher insulin, but not with higher glucose levels^11^. The second study reported that the same SNP exerts its effect also by generating either an intronic (CA)_16_ STR, which was associated with an increased risk for prediabetes, or a (CA)_15_ to (CA)_19_ STR, which were all related to a decreased prediabetes risk^12^. Recently, a rather small case-control study confirmed the association of rs10886471 with prediabetes and type 2 diabetes, as well as with so-called differentiated symptoms of diabetes^32^. However, the association of the SNP rs10886471 with type 2 diabetes in the first study was found to be specific for East Asians, and the second and third studies were performed in only a Chinese Hainan Island population. It is, therefore, not surprising that we found no significant association of the SNP rs10886471 with measurements of glucose metabolism in our European cohort. A possible explanation for the ethnic differences may be that the risk allele frequency of the SNP rs10886471 in East Asians is much higher than in Europeans (0.786 in the HapMap CHB population and 0.455 in the HapMap CEU population, respectively) (http://grch37.ensembl.org/Homo_sapiens/Info/Index). Of note, whether there was an association of the particular SNP with lipid metabolism was not investigated in either of these two studies.

Our study has some limitations. First, it is unclear whether our findings and conclusions can be applied to other populations. Since there is no other study in the literature investigating the effect of *GRK5* SNPs on cholesterol levels in humans, our results need replication in other ethnic groups. Second, our sample consisted of a cohort covering a wide range of age, adiposity, and insulin sensitivity. Thus, an impact of the SNPs in an older or a more insulin resistant population cannot be excluded. Finally, we studied only four common variants from selected gene regions and with MAFs ≥ 0.05, so that further SNPs in *GRK5* with possible effect on cholesterol levels may exist.

To summarize, in this study we report robust associations of two SNPs in *GRK5* gene with total and LDL-cholesterol as well as apoB levels. In our population, homozygotes for the minor (risk) allele had up to 20% higher cholesterol levels compared to homozygotes for the major allele. GRK5 may thus be involved in cholesterol metabolism. Furthermore, we detected a week association of one of the two SNPs with IMT. Although this association fell short from being statistically significant, it may be clinically relevant, because it is in line with data from *in vitro* and *in vivo* animal studies suggesting that GRK5 may have direct and indirect effects on atherosclerotic cardiovascular disease, and that apoB may be more predictive for the progression of cardiovascular disease than even oxidized LDL^33^. Thus, GRK5 represents a potential promising target for treatment strategies not only of total-, and particularly LDL-cholesterol levels, but possibly also of cardiovascular disease.

## Electronic supplementary material

Dataset 1

Supplementary material
