# Supplementary material for: Single Nucleotide Polymorphisms in the G-Protein Coupled Receptor Kinase 5 (GRK5) Gene are associated with Plasma LDL-Cholesterol Levels in Humans
Source: Sci Rep. 2018 May 17;8:7745. doi: 10.1038/s41598-018-26055-7 (PMC5958094; doi:10.1038/s41598-018-26055-7)
Supplement: Supplementary file 2 — Supplementary material [file 41598_2018_26055_MOESM2_ESM.pdf]

Single Nucleotide Polymorphisms in the G-Protein Coupled Receptor Kinase 5 (*GRK5*) Gene are associated with Plasma LDL-Cholesterol Levels in Humans

*

Stefan Z. Lutz, Mathias Falcenberg, Fausto Machicao, Andreas Peter, Martin Kächele, Elko Randrianarisoa, Angela Lehn-Stefan, Robert Wagner, Jürgen Machann, Fritz Schick, Martin Heni, Axel Ullrich, Andreas Fritsche, Norbert Stefan, Hans-Ulrich Häring, Harald Staiger, Konstantinos Kantartzis

Supplementary Info

Supplemental table 2 Associations of the SNPs rs9325562 and rs10886471 in *GRK5* gene with demographic and metabolic characteristics.

Parameter	rs9325562					rs10886471				
	**GG**	**GA**	**AA**	**p add**	**p dom**	**TT**	**TC**	**CC**	**p add**	**p dom**
n (females/males)	607 / 370	673 / 378	189 / 115	0.71#	0.46#	427 / 230	742 / 429	300 / 204	0.06#	0.21#
Age (years)	40.0 [29.0–50.0]	39.0 [29.0–51.0]	41.0 [30.3–51.8]	0.42	0.79	40.0 [30.0–50.0]	39.0 [29.0–51.0]	40.0 [29.0–51.0]	0.83	0.87
BMI ($kg \bullet m^{-2}$)	28.57 [24.40–36.02]	28.57 [24.05–35.81]	28.90 [24.36–35.17]	0.62	0.46	29.92 [24.39–37.03]	28.26 [24.14–35.36]	28.38 [24.48–34.98]	0.09	0.028
Waist circumference (cm)	96.0 [84.0–109.0]	95.0 [83.0–110.0]	96.0 [85.0–110.0]	0.93	0.91	97.5 [84.0–110.0]	94.0 [83.0–109.0]	95.8 [85.5–110.0]	0.26	0.09
Total body fat mass $_{MRT}$ (% BW)*	30.55 [22.79–37.49]	30.66 [23.97–38.55]	29.72 [23.18–35.31]	0.49	0.56	29.86 [24.86–38.46]	30.80 [24.18–37.90]	28.68 [21.39–35.24]	0.70	0.90
Visceral fat mass $_{MRT}$ (% BW)*	3.03 [1.88–4.27]	3.24 [1.93–4.37]	3.27 [1.90–4.49]	0.42	0.38	2.86 [1.81–3.81]	3.24 [2.09–4.49]	3.15 [1.88–4.77]	0.15	0.10
Liver fat $_{MRS}$ (%)**	3.27 [1.34–7.62]	3.43 [1.71–8.56]	3.54 [1.47–6.71]	0.88	0.35	3.25 [1.51–7.58]	3.31 [1.60–7.95]	3.88 [1.50–9.66]	0.70	0.81
Fasting glucose (mM)	5.11 [4.84–5.50]	5.11 [4.79–5.53]	5.17 [4.83–5.50]	0.99	0.78	5.17 [4.89–5.56]	5.11 [4.83–5.56]	5.11 [4.80–5.50]	0.11	0.40
2 h glucose $_{OGTT}$ (mM)	6.22 [5.22–7.22]	6.22 [5.17–7.28]	6.28 [5.28–7.39]	0.89	0.64	6.22 [5.31–7.28]	6.22 [5.17–7.28]	6.17 [5.17–7.16]	0.67	0.90
Fasting Insulin (pM)	61 [38–102]	60 [39–101]	63 [39–104]	0.55	0.48	64 [39–107]	58 [37–101]	63 [39–98]	0.40	0.75
2 h Insulin (pM)	365 [201–672]	357 [199–674]	364 [214–647]	0.69	0.66	365 [201–684]	356 [204–665]	364 [202–658]	0.49	0.47
Fasting free fatty acids (µM)	563 [421–725]	570 [434–723]	583 [439–730]	0.45	0.61	568 [422–726]	580 [434–734]	560 [422–712]	0.53	0.65
2 h free fatty acids (µM)	74 [46–115]	72 [47–116]	73 [48–108]	0.91	0.99	74 [47–116]	72 [46–111]	75 [48–122]	0.85	0.86
Fasting triglycerides (mg/dl)	101.0 [71.0–146.0]	103.5 [72.0–150.8]	101.0 [75.5–146.0]	0.13	0.044	104.0 [75.0–145.0]	99.0 [70.0–146.0]	107.5 [74.8–156.5]	0.24	0.68
Total cholesterol (mg/dl)	188.0 [166.0–214.0]	190.0 [167.0–218.0]	192.0 [165.0–221.5]	0.24	0.13	190.0 [168.0–216.0]	189.0 [166.0–216.0]	191.0 [166.0–218.0]	0.81	0.68
HDL-cholesterol (mg/dl)	51.0 [43.0–61.0]	52.0 [43.0–61.0]	52.0 [43.0–62.3]	0.44	0.32	52.0 [43.0–61.0]	52.0 [44.0–62.0]	51.0 [42.0–61.0]	0.49	0.85

LDL-cholesterol (mg/dl)	115.0 [95.0–136.0]	117.0 [96.0–140.0]	119.0 [97.0–139.0]	0.40	0.36	117.0 [98.0–141.0]	115.0 [94.0–136.0]	119.0 [96.0–140.0]	0.93	0.43
Apolipoprotein AI (mg/dl)	152.0 [135.0–170.0]	152.0 [134.0–173.0]	151.0 [133.0–167.0]	0.17	0.23	153.0 [135.5–169.0]	152.0 [135.0–172.0]	151.0 [131.0–171.0]	0.60	0.80
Apolipoprotein B (mg/dl)	89.0 [74.5–106.0]	91.0 [76.0–108.0]	91.0 [75.0–108.0]	0.47	0.29	90.0 [76.0–107.0]	89.0 [74.0–107.0]	92.0 [75.0–107.0]	0.62	0.57
Glycated hemoglobin - HbA1c (%)	5.40 [5.10–5.70]	5.40 [5.10–5.70]	5.40 [5.10–5.80]	0.57	0.80	5.40 [5.10–5.70]	5.40 [5.10–5.70]	5.40 [5.10–5.70]	0.82	0.74
HOMA-IR index (10^{-6} mol•U•l^{-2})	2.31 [1.41–4.00]	2.27 [1.41–4.04]	2.40 [1.45–4.04]	0.59	0.56	2.45 [1.46–4.22]	2.21 [1.38–4.02]	2.38 [1.42–3.79]	0.64	0.91
Insulin sensitivity $_{OGTT}$ (10^{19} l^2•mol^{-2})	10.63 [6.37–17.45]	10.81 [6.17–17.69]	10.04 [6.57–17.23]	0.77	0.74	10.23 [6.26–17.12]	10.97 [6.44–17.72]	10.49 [6.24–17.98]	0.62	0.82
ISI_{clamp} (10^6 l•kg^{-1}•min^{-1})***	0.075 [0.052–0.111]	0.073 [0.047–0.111]	0.074 [0.051–0.109]	0.57	0.31	0.075 [0.051–0.113]	0.073 [0.048–0.107]	0.077 [0.047–0.119]	0.13	0.07
C-peptide 30’(pmol/l)	1902 [1442–2504]	1875 [1405–2524]	1890 [1476–2429]	0.43	0.47	1897 [1489–2499]	1870 [1408–2455]	1953 [1461–2580]	0.67	0.34
IGI [($Ins_{30'}$-$Ins_{0'}$)/($Glc_{30'}$-$Glc_{0'}$)]	143.3 [89.1–226.7]	140.6 [85.2–232.2]	131.9 [81.7–191.2]	0.07	0.24	145.7 [89.8–238.6]	138.4 [81.8–220.2]	138.2 [91.1–229.0]	0.75	0.24
AUC C-$Pep_{0'-30'}$/AUC $Glc_{0'-30'}$	185.1 [144.3–242.0]	186.3 [142.6–240.5]	179.8 [146.3–229.2]	0.26	0.58	187.0 [146.8–240.7]	182.4 [140.6–234.3]	188.5 [146.6–248.4]	0.34	0.55
AUC C-.$Pep_{0'-120'}$/AUC $Glc_{0'-120'}$	293.1 [245.2–369.2]	303.5 [240.6–367.4]	298.3 [243.8–354.6]	0.53	0.73	301.8 [245.1–367.9]	293.3 [239.2–360.6]	305.3 [246.3–373.4]	0.42	0.56

*available in 339 subjects; **available in 455 subjects, ***available in 498 subjects.

Values represent means ± SE (standard error). For statistical analyses, non-normally distributed parameters were log transformed. The genotype effect was tested using additive and dominant inheritance models. BMI, waist circumference, total body fat mass, visceral fat mass and liver fat were adjusted for age and gender. Glucose and insulin levels, glycated hemoglobin, and insulin sensitivity measures (HOMA-IR, insulin sensitivity from the OGTT and ISI_{clamp}) were additionally adjusted for BMI. Lipid parameters (free fatty acids, total-, HDL- and LDL-Cholesterol, and triglycerides) were further adjusted for lipid-lowering medication. Insulin secretion measures (C-peptide 30’, IGI, AUC C-peptide 0-30 / AUC Glc 0-30, AUC C--peptide 0-120/ AUC Glc 0-120) were adjusted for gender, age, BMI and insulin sensitivity from the OGTT. #χ2-test. BW, body weight; ISI, insulin sensitivity index; IGI, insulinogenic index; Ins, insulin; C-Pep, C-peptide; Glc, glucose; AUC, Area Under the Curve.

Supplemental table 3 Associations of rs10466210 and rs1980030 with plasma lipids after excluding subjects with BMI ≥ 65 kg/m^2

	**rs10466210**		**rs1980030**	
Variable	**p additive**	**p dominant**	**p additive**	**p dominant**
Fasting triglycerides (mg/dl)	0.31	0.33	0.046	0.026
Total cholesterol (mg/dl)	0.0015	0.0014	0.0043	0.0036
HDL-cholesterol (mg/dl)	0.18	0.12	0.71	0.91
LDL-cholesterol (mg/dl)	0.0033	0.0055	0.01	0.035
Apolipoprotein AI (mg/dl)	0.17	0.09	0.38	0.72
Apolipoprotein B (mg/dl)	0.0027	0.0021	0.014	0.031

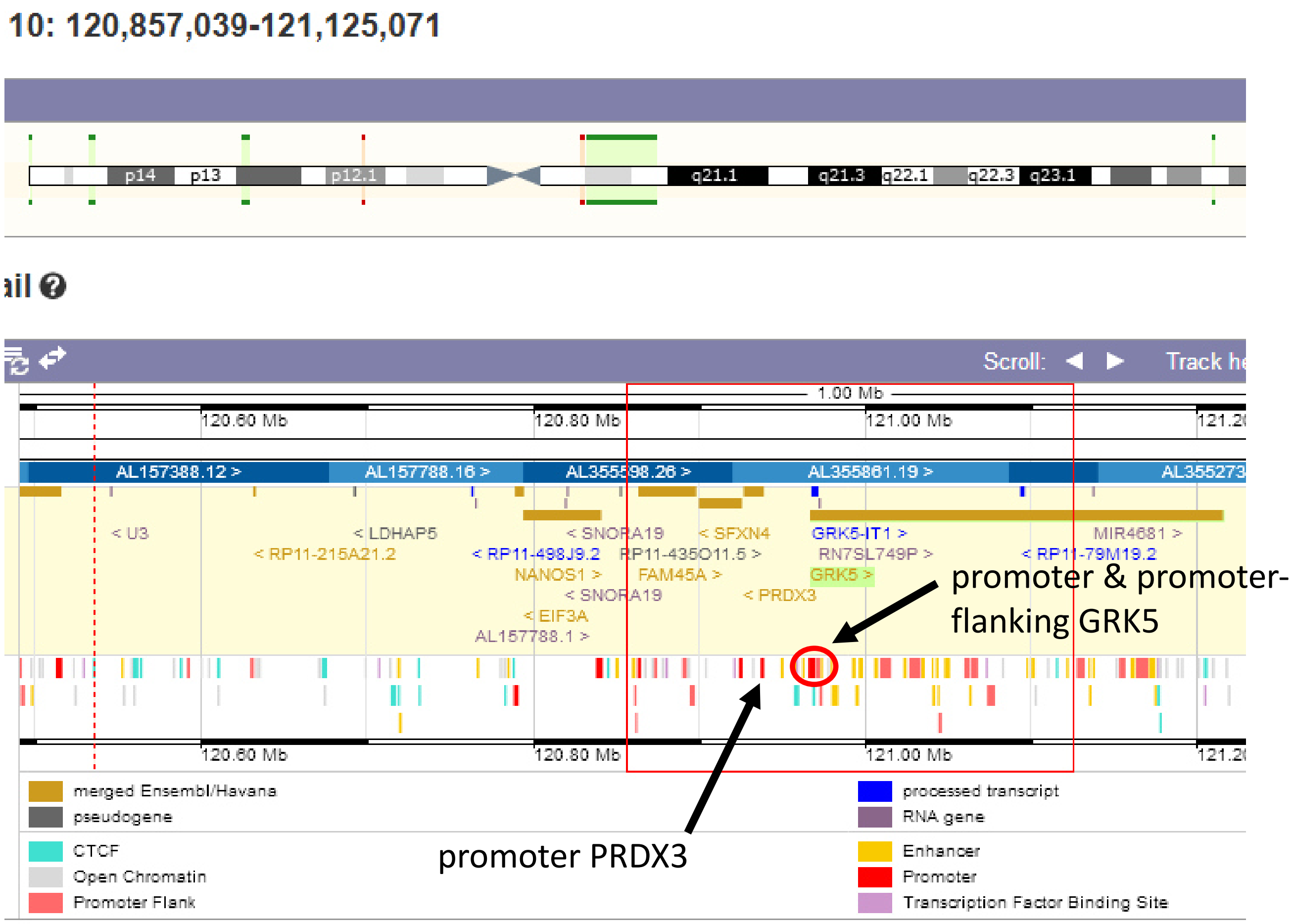

Supplemental Figure 1. Details on regulatory elements in the genomic locus encompassing *GRK5* and its upstream flanking genes *PRDX3* and *SFXN4*. Data are presented as displayed by the Ensembl 1000 Genomes browser (http://grch37.ensembl.org/Homo_sapiens/Info/Index). The promoter (in red) and promoter-flanking regions (in pink) of the *GRK5* gene (marked by a red circle) are clearly separated from the promoter regions of the adjacent genes.

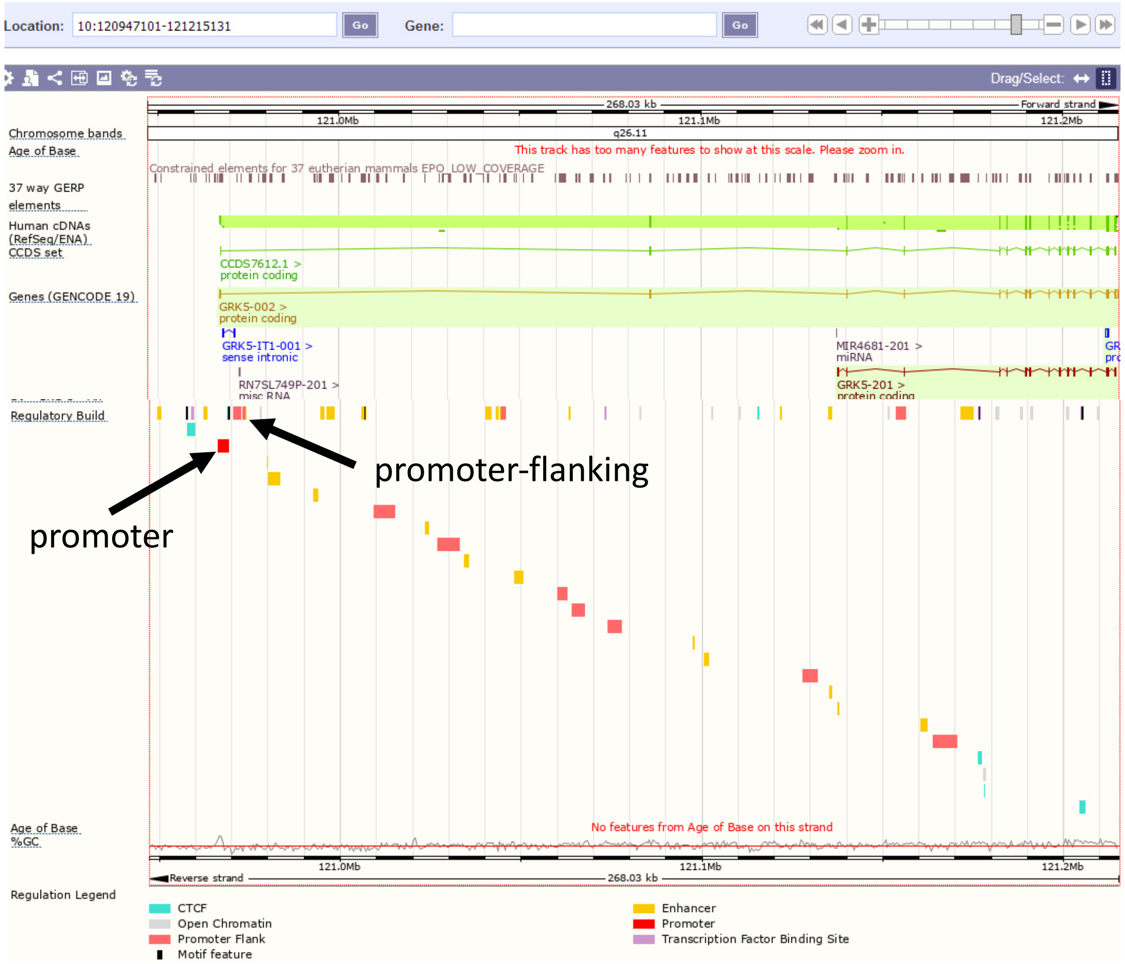

Supplemental Figure 2. Gene + 20 kb 5'-flanking region. Zoomed picture with details on regulatory elements in the promoter and gene regions of *GRK5*. Data are presented as displayed by the Ensembl 1000 Genomes browser (http://grch37.ensembl.org/Homo_sapiens/Info/Index). The promoter (in red) and its promoter-flanking regions (in pink) are clearly separated from other putative promoter-flanking regions (in pink) more downstream. The role of the latter is uncertain due to their distance to the core promoter.
